# TiO_2_ immobilized on 2D mordenite: effect of hydrolysis conditions on structural, textural, and optical characteristics of the nanocomposites

**DOI:** 10.3762/bjnano.16.12

**Published:** 2025-02-10

**Authors:** Marina G Shelyapina, Rosario Isidro Yocupicio-Gaxiola, Gleb A Valkovsky, Vitalii Petranovskii

**Affiliations:** 1 Department of Nuclear Physics Research Methods, Saint-Petersburg State University, 7/9 Universitetskaya nab., St. Petersburg 199034, Russia; 2 Tecnológico Nacional de México/Instituto Tecnológico Superior de Guasave, Carretera a La Brecha Sin Número, Ejido Burrioncito, Guasave 81149, Sin., Mexico; 3 Center for Nanoscience and Nanotechnology, National Autonomous University of Mexico (CNyN, UNAM), Ensenada, Baja California 22860, Mexico

**Keywords:** 2D zeolite, hierarchical porosity, mordenite, TEOT, TiO_2_, titanium tetraethoxide

## Abstract

A series of novel TiO_2_/2D mordenite nanocomposites were synthetized by the introduction of titanium tetraethoxide (TEOT) into the interlamellar space of 2D mordenite, its subsequent hydrolysis in water or a solution of 70% ethanol in water for 6, 12, and 24 h, and calcination. The resulting TiO_2_/2D mordenite materials were studied by a set of complementary characterization techniques, including XRD, SEM-EDX, TGA, N_2_ sorption, NMR, XPS and UV–vis spectrometry. It was observed that treatment in 70% ethanol solution preserves the ordered layered structure of 2D mordenite because TEOT hydrolysis is slowed down. This, in turn, leads to higher mesoporosity after calcination due to anatase nanoparticles of about 4 nm preventing the collapse of the interlamellar space. Immobilization of TiO_2_ on the zeolite surface is evidenced by the formation of Si–O–Ti bonds. The bandgap width of the synthetized nanocomposites was found to be sensitive to the hydrolysis medium.

## Introduction

Zeolites are important heterogeneous catalysts in various industrial processes. More and more functional materials based on zeolites are being searched for, including zeolites with hierarchical micro- and mesoporosity [1–6]. Zeolites with hierarchical porous structures can be synthesized using different strategies [4], for example, aggregation of nanocrystals, use of templates, and creation of mesoporosity by forming pillars between 2D lamellae of zeolites separated by cetyltrimethylammonium bromide (CTAB) layers. The latter method involves the synthesis of layered 2D zeolites in the presence of CTAB and organic structure directing agents (OSDAs), followed by calcination to remove them from the resulting products [1].

The choice of the OSDA determines not only the interlamellar distance and, hence, the mesopore size, but also the aluminum distribution and acidic properties of the obtained material, which are key parameters for catalysts [5,7–10]. One widely used component for the synthesis of 2D zeolites is CTAB [5,9,11–13].

To prepare mesoporous materials from hybrid zeolite–CTAB aggregates, inorganic pillars are pre-formed in the CTAB layers, which will keep the zeolite lamellae from collapsing when burning the organic phase. The flexibility in the choice of material to form pillars creates a wide range of potential new materials for targeted applications. Typically, such pillars are amorphous SiO_2_ nanoparticles formed during the hydrolysis of tetraethoxysilane (TEOS) introduced into liquid crystalline 2D CTAB layers that fill the interlamellar space between the 2D zeolite nanosheets. Tetraethoxytitanium (TEOT) is a homolog of TEOS, and its hydrolysis similarly leads to the formation of TiO_2_.

TiO_2_ is a well-known photocatalyst whose efficiency depends on a number of factors, including the crystalline phase, particle size, and degree of crystallinity. The most active phase of TiO_2_ is considered to be anatase. Its nanoparticles usually show higher efficiency than the bulk phase, but the bandgap of anatase particles smaller than 10 nm is very sensitive to their size [14].

One of the disadvantages of such free photocatalyst nanoparticles is the limitation of mass transfer between solid and liquid phases. From this perspective, the problem of immobilization of TiO_2_ nanoparticles is of great importance. Many materials are considered as a possible support for immobilization, including porous ceramics [15], glass [16–17], porous carbon materials [18–19], mesoporous silica [20–23], and zeolites [24–27]. Recent studies have confirmed that direct synthesis of TiO_2_ in mesoporous silica or zeolites provides strong immobilization of TiO_2_ nanoparticles through Ti–O–Si bonding [21–23].

Previously, we reported the results of the trial synthesis of a new TiO_2_/2D mordenite nanocomposite [28]. The material was obtained from a composite consisting of lamellar mordenite separated by CTAB layers through the substitution of TEOS for TEOT and subsequent hydrolysis. As a result, the pillars separating the mordenite layers were not made of silica as in the routine synthesis method for the preparation of mesoporous materials, but of anatase nanoparticles of about 4 nm in size. It was found that the textural properties of the resulting materials are sensitive to the environment in which TEOT hydrolysis takes place.

Alkoxides are, generally, not stable in protic solvents such as water. However, because of this property they are widely used as starting products in a large number of reactions. The hydrolysis of various metal alkoxides by pure water, or its mixtures with primarily alcohols or other solvents, are the basis of the sol–gel method to obtain oxide materials. The tendency of metal alkoxides to this reaction can be considered as their most important chemical property. For various reasons, the sol–gel method is mainly associated with the hydrolysis of Si(OR)_4_; this reagent is readily available, inexpensive, and its hydrolysis proceeds relatively smoothly, as discussed in numerous original papers and reviews [29]. However, the hydrolysis of M(OR)_4_ (M = metal), unlike the hydrolysis of Si(OR)_4_, is an extremely fast process. Thus, the basic concepts that were developed specifically for Si(OR)_4_ cannot be applied to the hydrolysis of any arbitrary metal alkoxides. The higher coordination number of metals in their alcoholic and hydroxy derivatives compared to Si(OR)_4_ leads to a high propensity for oligomerization and polymerization of metal alkoxides after the first stages of hydrolysis.

In the case of tetraethoxysilane, the overall reaction can be written as [29]:


[1] $$\text{Si}{\left(\text{OEt}\right)}_{4}+2{\text{H}}_{2}\text{O}\Rightarrow {\text{SiO}}_{2}+4\text{HOEt}.$$

The situation in the case of titanium alkoxides is more ambiguous. In a calorimetric hydrolysis study of Ti(OR)_4_ at different concentrations, the values of reaction enthalpy of the first hydrolysis stage were measured for R = Et, iPr, and *n*-Bu [29]. It turned out that further hydrolysis proceeds much slower and with very little heat release (for R = Et its value is zero within the accuracy of the experiment).


[2] $$\text{Ti}{\left(\text{OR}\right)}_{4}+{\text{H}}_{2}\text{O}=\text{Ti}{\left(\text{OR}\right)}_{2}\left(\text{OH}\right)+\text{ROH}$$

Therefore, this step is immediately followed by condensation:


[3] $$n\text{Ti}{\left(\text{OR}\right)}_{3}\left(\text{OH}\right)\Rightarrow {\text{Ti}}_{n}{\text{O}}_{x}{\left(\text{OR}\right)}_{4n-2x}.$$

The final composition of the hydrolysis products of alkoxides of titanium roughly corresponds to TiO_1.5_(OR)·*y*ROH, where *y* = 0.15–1.00 depending on the nature of the alcohol. The residual carbon during thermal treatment in air is eliminated in the process of crystallization at 400–550 °C; thus, titanium dioxide nanoparticles are obtained [29].

In this study we investigate in detail the influence of the hydrolysis medium and the duration of the hydrolytic process on composition, local structure, morphology, texture, and optical properties of TiO_2_/2D mordenite nanocomposites.

## Results and Discussion

TiO_2_/2D mordenite compounds were synthetized from the parent layered MOR-L by introduction of TEOT, its further hydrolysis, and calcination. Further details are given in the Experimental section. The calcined (C) samples are labeled as Ti-W*N*h-C and Ti-E*N*h-C with *N* = 6, 12, and 24 for materials hydrolyzed in water (W) and 70% ethanol solution (E) for 6, 12, and 24 h, respectively. The non-calcined samples are designated as Ti-W*N*h and Ti-E*N*h.

### XRD, ^27^Al NMR, and SEM-EDX studies

**Figure 1:**
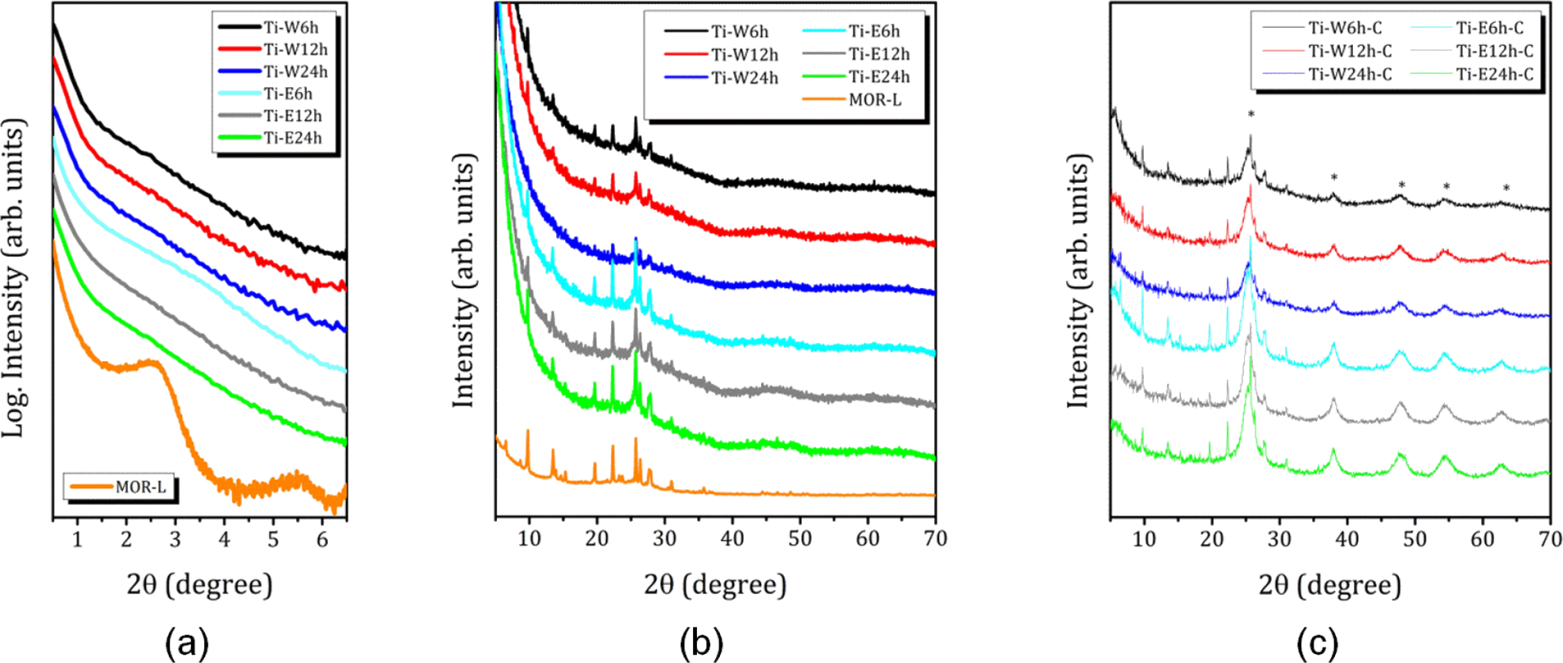
(a) Small-angle powder patterns for the parent compound MOR-L and the samples after TEOT hydrolysis. (b, c) Full XRD powder patterns for the Ti-loaded samples after (b) TEOT hydrolysis and (с) subsequent calcination. Asterisks indicate the anatase structure formed after calcination.

Figure 1a and Figure 1b show small-angle and full X-ray diffraction (XRD) patterns, respectively, for the samples containing hydrolyzed forms of Ti after hydrolysis of TEOT. The full XRD patterns of the hydrolyzed samples after calcination are shown in Figure 1c. For comparison, the corresponding patterns for the parent compound MOR-L are also given.

As it was shown in our previous study [5], the as-synthetized lamellar MOR-L exhibits peaks characteristic of a 3D mordenite structure and small-angle peaks at 2θ = 2.7° and 5.5°, which correspond to the (001) and (002) peaks of the ordered layered structure with an interplanar distance of 3.2 nm. According to Figure 1b, the introduction of TEOT and its further hydrolysis do not significantly perturb the mordenite peaks. But the small-angle reflection peaks corresponding to the lamellar structure disappear (Figure 1a); traces of a blurred peak are observed only for the sample with minimal hydrolysis time in 70% ethanol solution. An obvious reason for the loss of lamellar structure is the rate of hydrolysis, which is much higher for TEOT than for TEOS [5]. The rapid and apparently uneven growth of oligomers in different regions of the interlamellar layers leads to a disruption of the long-range order of the lamellae.

In addition, the full XRD patterns evidence the formation of an amorphous phase, presumably nanosized TiO_2_ (Figure 1b). Zang et al. [30] showed that the XRD pattern of nanometer-sized amorphous TiO_2_ consists of broad humps at 2θ values of 35°, 53°, and 75°. In the TiO_2_/2D mordenite composites studied in our work, the first hump is masked by the main zeolite peaks, but the remaining two are identical to the ones reported in [30]. Amorphous TiO_2_ particles exhibiting such an XRD pattern can be described as small strained anatase-like crystalline cores with strongly distorted shells. These amorphous nanoparticles are responsible for the formation of single-phase nanocrystalline anatase upon heating [30]. This is exactly what we observe. Figure 1c shows that, after calcination, additional large peaks appear in all samples, which can be attributed to anatase TiO_2_ nanoparticles. The average size of the TiO_2_ nanoparticles, as determined from single diffraction peaks of experimental patterns using the Scherrer formula, is about 4.0 nm for the composites prepared by hydrolysis in water, and slightly larger, about 4.3 nm, after hydrolysis in 70% ethanol solution (Table 1).

**Table 1:** Average TiO_2_ particle size ⟨*d*⟩ and elemental composition, determined by EDX and XPS, of the as-synthetized and Ti-loaded samples, and their TiO_2_ content.

Sample	Method	Si/Al	Na/Al	Ti/Al	TiO_2_ (wt %)	⟨*d*⟩ (nm) XRD	Ref.

MOR-L	EDX	8.4 ± 0.3	1.14 ± 0.03	—	—	—	[5]
Ti-W6h-C	EDX	10.2 ± 0.4	0.5 ± 0.1	16 ± 1	63 ± 1	4.0 ± 0.1	[28]
	XPS	6.2 ± 0.1	ND^a^	7.6 ± 0.3	52 ± 1		
Ti-W12h-C	EDX	10.2 ± 0.1	0.2 ± 0.1	29 ± 1	73 ± 1	4.1 ± 0.1	this paper
	XPS	4.6 ± 0.1	ND^a^	6.8 ± 0.3	60 ± 1		
Ti-W24h-C	EDX	10.9 ± 0.2	0.4 ± 0.1	30 ± 1	72 ± 1	3.9 ± 0.1	this paper
	XPS	4.8 ± 0.1	ND^a^	8.2 ± 0.3	63 ± 1		
Ti-E6h-C	EDX	9.3 ± 0.6	0.3 ± 0.2	15 ± 2	61 ± 2	4.2 ± 0.1	[28]
	XPS	6.9 ± 0.1	ND^a^	7.0 ± 0.3	51 ± 1		
Ti-E12h-C	EDX	10.6 ± 0.2	0.3 ± 0.1	20 ± 1	66 ± 1	4.3 ± 0.1	this paper
	XPS	4.2 ± 0.1	ND^a^	10.5 ± 0.3	70 ± 1		
Ti-E24h-C	EDX	10.5 ± 0.2	0.4 ± 0.1	10 ± 1	50 ± 1	4.4 ± 0.1	this paper
	XPS	5.3 ± 0.1	ND^a^	6.3 ± 0.3	55 ± 1		

^a^The Na 1s peak overlaps with the Ti LMM Auger peak, which impedes correct evaluation of the Na content.

In addition, it should be noted that calcination, independent of the medium of TEOT hydrolysis, leads to the complete disappearance of the long-range ordering of the zeolite lamellae (no peak at 2θ < 5°; the SAXS patterns are not shown here). This is an important difference between TEOS and TEOT, studied in our previous work [5], for which even after hydrolysis for 12 h in water the long-range order of the lamellae was preserved.

The results of the elemental analysis using energy dispersive X-ray (EDX) and X-ray photoelectron spectroscopy (XPS) of the parent lamellar sample and TiO_2_-loaded samples are summarized in Table 1. Upon formation of TiO_2_ nanoparticles, a partial dealumination of mordenite occurs. However, the Al distribution over the sample depth is not homogenous. XPS shows that Al is accumulated on the sample surface. The hydrolysis medium does not significantly affect the total Si/Al ratio, but hydrolysis reactions longer than 6 h result in a higher aluminum concentration on the surface. It should be also noted that all TiO_2_-loaded samples are characterized by low sodium content. We remind the readers that Na^+^ is a charge compensating cation; for an ideal sodium zeolite, Na/Al = 1. The parent MOR-L compound even exhibits a small excess of positive charge (Na/Al > 1) that is balanced by Br^−^ anions [5]. In the process of post-hydrolysis calcination, the removal of CTAB results in the creation of protonated centers, and the role of compensating cations is eventually passed to protons.

According to the elemental analysis data (Table 1), the TiO_2_ loading exceeds 50 wt % in all studied composites. Comparing the data of methods with different measuring depths (i.e., EDX and XPS), we can conclude that Ti is more or less uniformly distributed over the depth in the presented samples. This allows us to state that TiO_2_ nanoparticles not only accumulate on the sample surface but are also present in the interlamellar space, which correlates with the nitrogen adsorption data reported next. For both hydrolysis media, the maximum TiO_2_ content is reached after 12 h of treatment. For Ti-E24h-C, the lower TiO_2_ content compared to TI-E12h-C can be attributed to the formation of 3D mordenite fibers, clearly visible in the SEM images (see below in Figure 3).

From the comparison of the surface (XPS) and volume (EDX) content of TiO_2_, we can not only deduce a fairly uniform distribution of titania over the volume, but also a somewhat higher volume content for samples obtained by hydrolysis in water. This suggests that the formation of anatase particles occurs both on the surface and in the entire accessible mesoporous volume, creating titania pillars for the mesoporous system.

**Figure 2:**
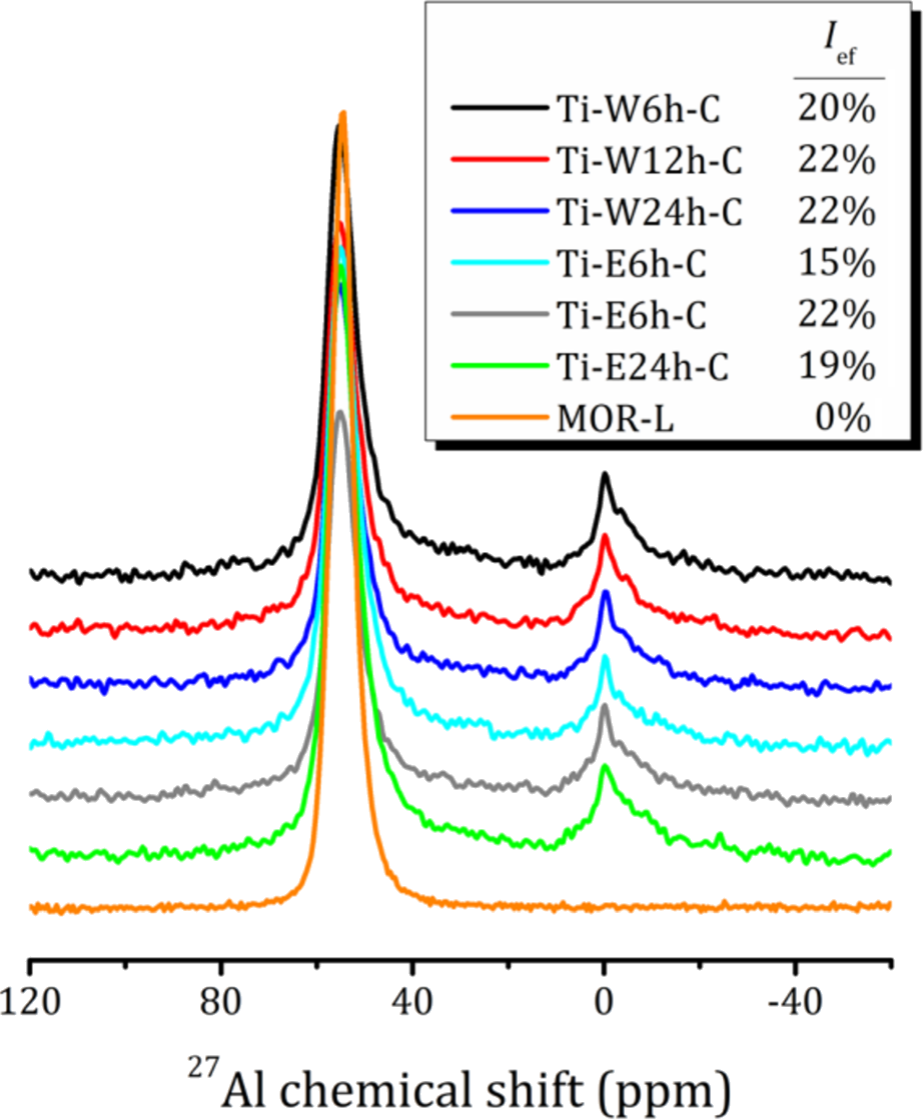
^27^Al MAS NMR spectra of the studied samples. The relative integral intensity of the line at 0 ppm corresponding to extra-framework Al species *I*_ef_ is shown in the legend.

Figure 2 shows the ^27^Al magic angle spinning nuclear magnetic resonance (MAS NMR) spectra of the parent compound MOR-L and the TiO_2_-loaded samples. They confirm the regularity of the zeolite frameworks of the as-prepared samples. The spectrum consists of only one line at 54 ppm, which corresponds to aluminum in regular tetrahedral sites of the zeolite framework. As it was reported earlier for 2D mordenite pillared by amorphous SiO_2_, obtained by hydrolysis of TEOT in water, the removal of CTAB leads to a partial removal of Al from the zeolite frameworks [5]. The same is observed for the TiO_2_-loaded samples. An additional line at about 0 ppm corresponds to extra-framework six-coordinated Al species [31–34], and the relative content of this extra-framework Al does not depend much on duration or medium of hydrolysis (about 20% for all samples except Ti-E6h-C, for which it is 15%) and is mainly determined by the mutual arrangement of the CTA^+^ cations and Al in the zeolite lamellae in the parent MOR-L compound. The CTA^+^ cations are localized near [AlO_4_]^−^ tetrahedra; the removal of organics upon calcination results in a partial collapse of the local zeolite structure.

**Figure 3:**
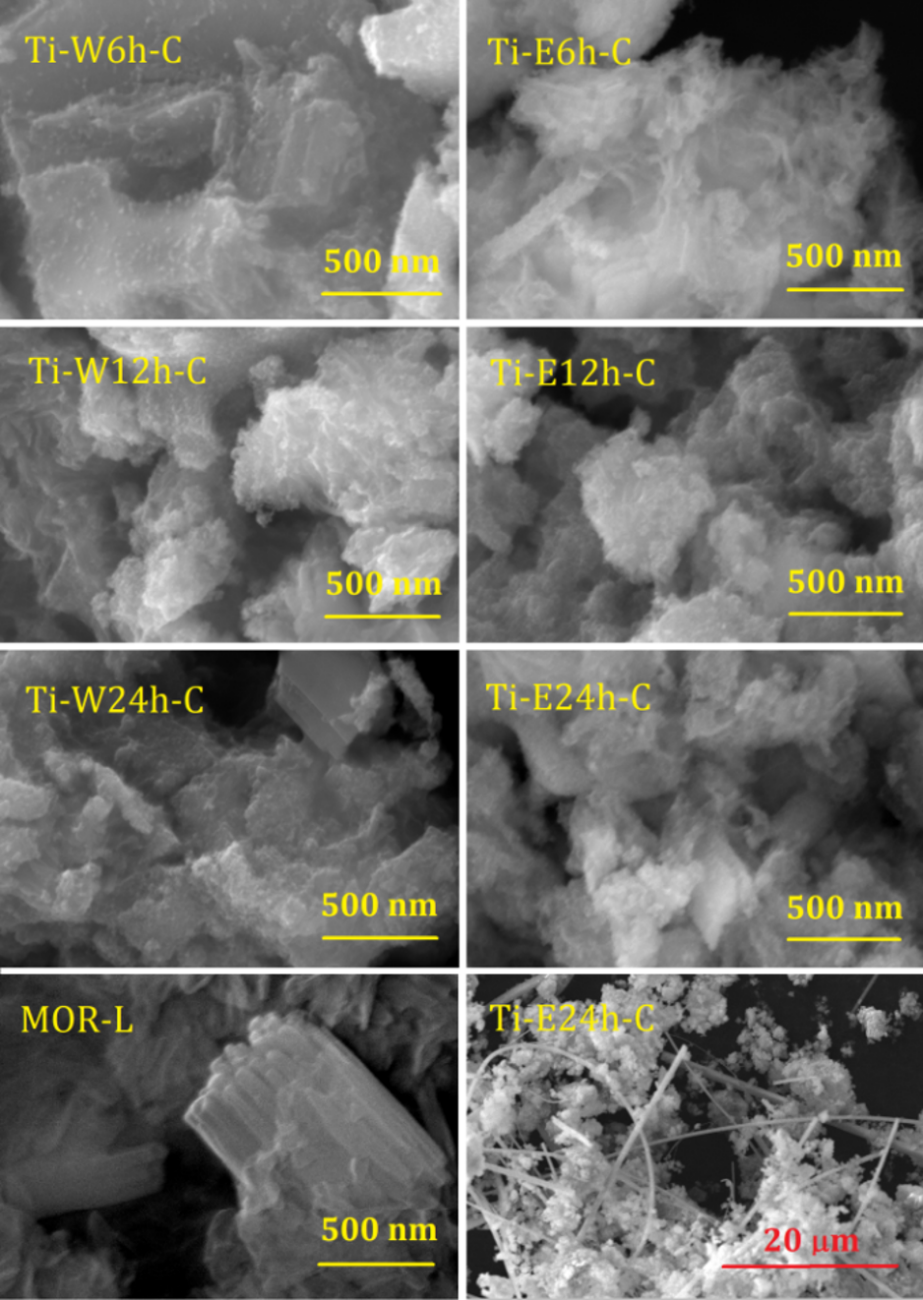
SEM images of the parent lamellar mordenite MOR-L (bottom left) and TiO_2_-loaded samples. The bottom-right image shows the formation of mordenite crystals in the Ti-E24h-C sample.

Figure 3 shows images of all studied composites. The scanning electron microscopy (SEM) image of the initial lamellar mordenite sample MOR-L is also shown for comparison. As can be seen, MOR-L exhibits elongated plates up to 1 μm long and 0.1 μm wide, combined into stacks. After introduction of TEOT followed by hydrolysis and calcination, all composites have a similar morphology. Thin plates of about 0.5 μm in size covered with nanoparticles of about 10 nm in size. The samples obtained by hydrolysis in 70% ethanol solution exhibit a more foam-like shape. Moreover, hydrolysis in 70% ethanol solution for 24 h results in the formation of thin single-crystalline fibers of mordenite.

### Al 2p, Ti 2p, and O 1s XPS studies

**Figure 4:**
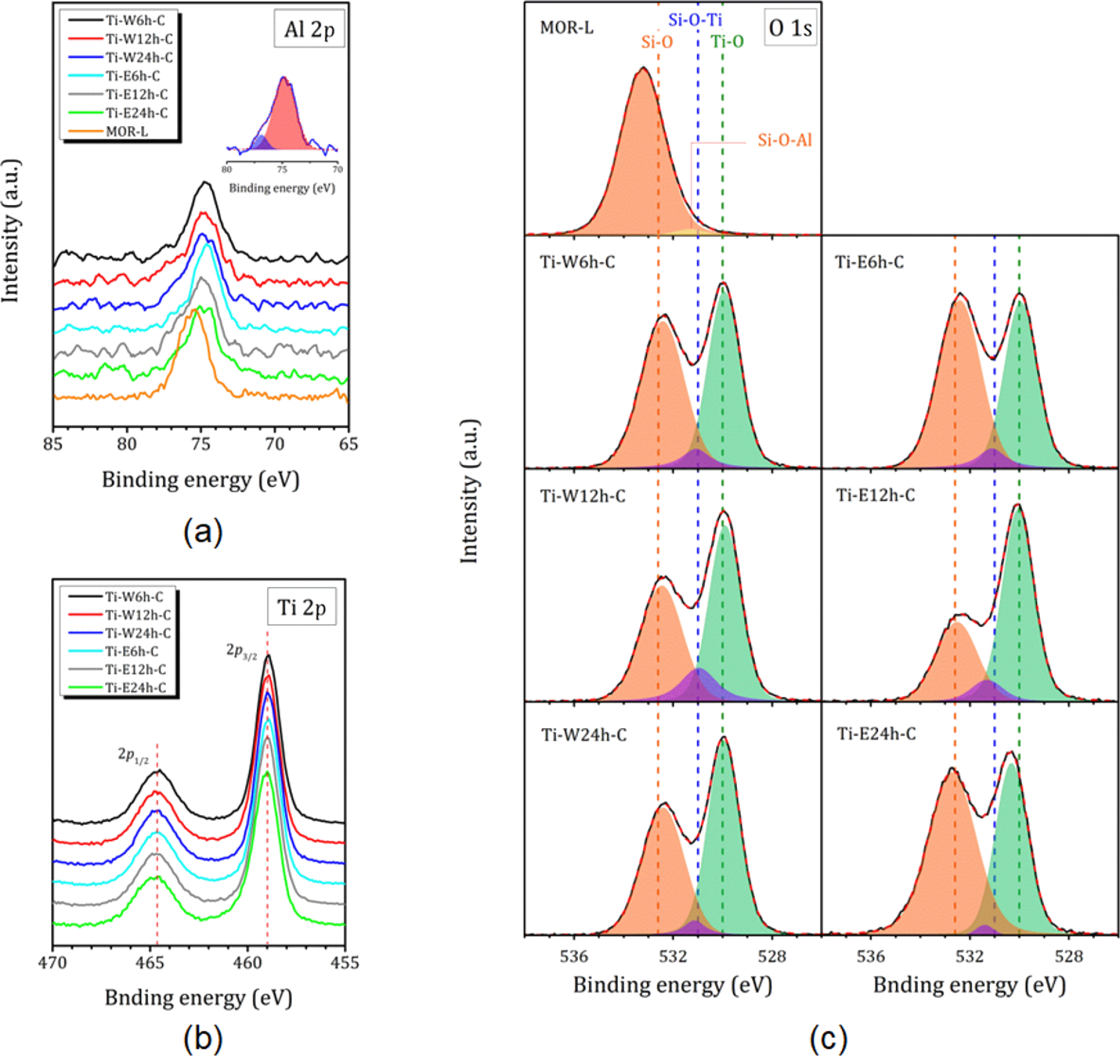
(a) Al 2p, (b) Ti 2p, and (c) decomposed O 1s XPS spectra of the Ti-loaded samples. The Al 2p and O 1s XPS spectra of the MOR-L compound are given for comparison. The inset in (a) shows the decomposition of the Al 2p spectrum for Ti-W24h-C.

The XPS spectra of Al 2p, Ti 2p, and O 1s for the composites are shown in Figure 4. Figure 4a and Figure 4c also show the Al 2p and O 1s spectra of the starting MOR-L compound. No perturbation is observed in the Si 2p region. The only peak at 104.0 eV, corresponding to Si 2p in MOR-L, is slightly shifted towards a lower binding energy (103.3 eV) for all Ti-loaded samples.

The Al 2p XPS spectra of the Ti-loaded composites show differences compared to the initial MOR-L compound (peak at 75.5 eV). The peak is shifted towards lower binding energies (74.6–74.8 eV), and a shoulder appears at 77.5–76.9 and 76.8–76.4 eV for Ti-W*N*h-C and Ti-E*N*h-C, respectively. An exemplary decomposition of the Al 2p XPS spectrum for Ti-W24h-C is shown in the inset of Figure 4a. For the Ti-W*N*h-C series, the position of the additional peak strongly depends on the duration of hydrolysis (shift towards lower binding energy with increasing hydrolysis time), whereas the duration of hydrolysis in 70% ethanol solution has no significant effect on the position of the additional peak, but only on its intensity. This peak can be associated with extra-framework Al species, which according to ^27^Al NMR amount to a total of about 20% (Figure 2). For Ti-E24h-C, a low intensity peak appears at 72.7 eV. This peak can be related to framework Al in the 3D mordenite fibers, which are clearly seen in the SEM image (Figure 3). It should be noted that very often in zeolites the peak at 74.5–74.8 eV is attributed to the presence of Al_2_O_3_ [35–36]; however, as it will be shown further, this contradicts to the ^27^Al NMR spectra.

The Ti 2p XPS spectra of the Ti-W*N*h-C and Ti-E*N*h-C samples show peaks corresponding to Ti 2p_1/2_ (464.6 eV) and Ti 2p_3/2_ (459.0 eV) transitions, indicating that Ti is in the Ti(IV) state [37] (Figure 3b). To identify the interactions between TiO_2_ nanoparticles and zeolite layers in the composites, O 1s XPS spectra were analyzed (Figure 4c). The starting compound MOR-L shows a characteristic peak of Si−O bonds (533.2 eV) with a small shoulder at 531.3 eV, which can be attributed to Si−O−Al. The introduction of TiO_2_ shifts the Si−O peak down to 532.4–532.6 eV, and a characteristic peak of Ti−O bonds at 529.9 eV appears (for samples hydrolyzed in 70% ethanol solution for 24 h, it is shifted towards 530.3 eV). The signals from Al–O and Al–OH, expected at 532 and 533 eV, respectively, are evidently masked by a broad signal from the Si–O bonds. The O 1s spectra also reveal the emergence of a new O state at about 530.9–531.2 eV, which can be assigned to the formation of Si–O–Ti bonds [27]. A decrease of the Si–O–Ti signal with the hydrolysis duration suggests that the shortest hydrolysis time provides the most efficient immobilization of TiO_2_ nanoparticles on zeolites sheets. However, this conclusion is difficult to confirm quantitatively as Si−O−Al bonds also may provide a contribution to this line.

### Nitrogen sorption and thermogravimetric studies

**Figure 5:**
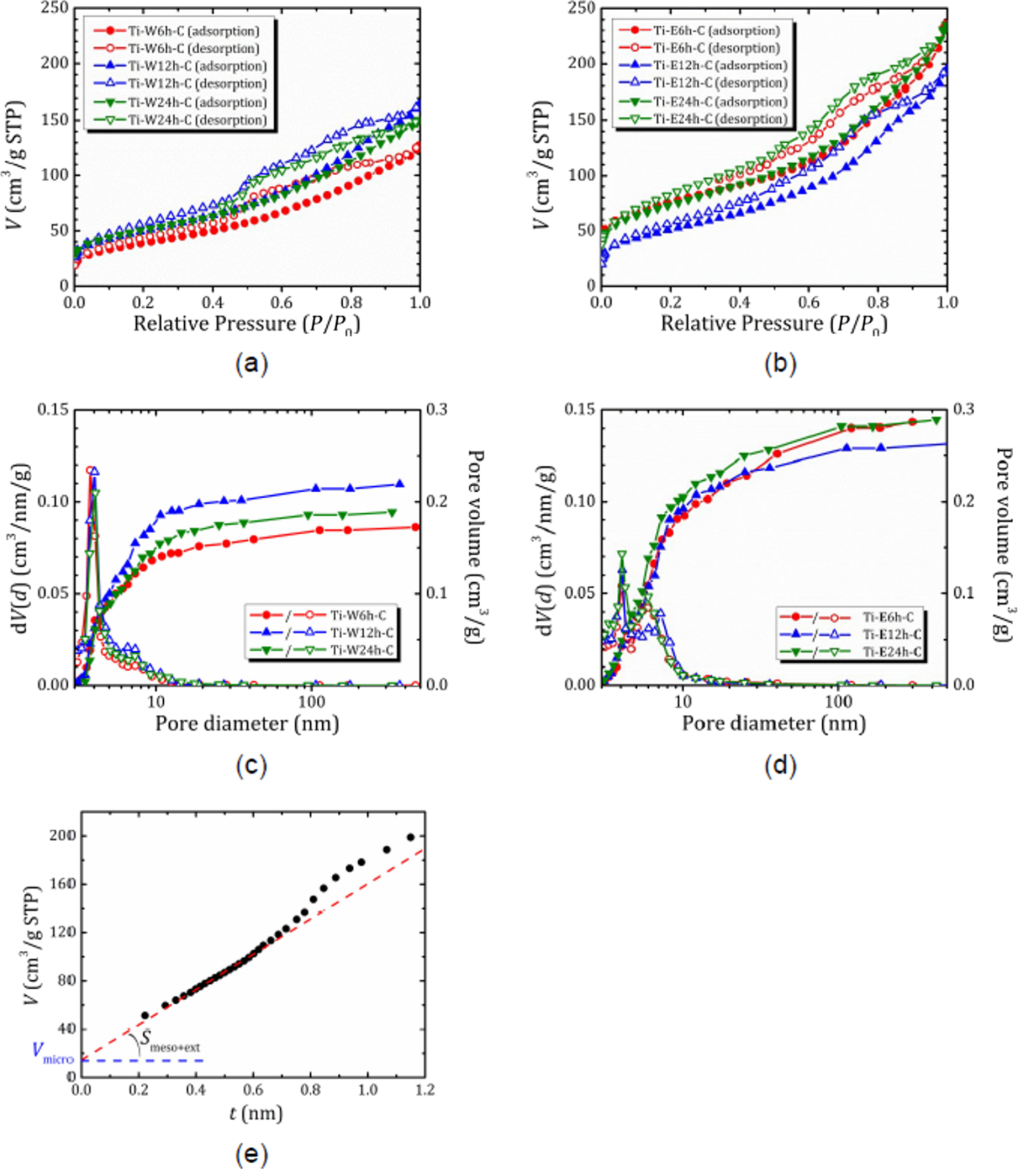
(a, b) Nitrogen adsorption isotherms at 77 K, (c, d) pore size distribution and pore volume in calcined nanocomposites Ti-W*N*h-C (a, c) and Ti-E*N*h-C (b, d); (e) *t*-plot for Ti-E6h-C with a linear fit in the *t* range from 0.33 to 0.6 nm.

Figure 5a,b shows the N_2_ adsorption/desorption isotherms of the studied nanocomposites. They demonstrate features characteristic of hierarchical porous structures possessing both micro- and mesoporosity. At low pressure, there is a sharp gas absorption (isotherm I or II according to IUPAC classification); at increasing pressure, the absorption continues and is accompanied by a hysteresis loop (isotherm IV according to IUPAC classification). For the series of Ti-W*N*h-C samples hydrolyzed in pure water, the inflection point on the desorption curves appears around *P*/*P*_0_ = 0.5, and it is followed by a broad hysteresis loop. The shape of this loop mainly corresponds to bottle-shaped pores (type H2, more precisely H2b, which corresponds to a pore blocking effect but without percolation, which may also indicate a narrow size distribution of pore cavities), with some presence of slit-shaped pores (type H3). Hydrolysis in 70% ethanol solution (i.e., Ti-E*N*h-C series) results in a wider distribution of mesopores by size. Volume *V*_BJH_ and diameter *D*_BJH_ of the mesopores determined from the desorption branch of the hysteresis loops are given in Table 2. The surface area *S*_BET_ of the samples were calculated using adsorption data in the range of relative pressures of 0.07–0.22. In addition, to estimate micropore surface area and volume, we applied the *t*-plot method, which plots the adsorbed volume as a function of the effective thickness (*t*) of the adsorbed layer. For a correct transformation of relative pressure (*P*/*P*_0_) to *t,* we used the formulas proposed in [38–39] for hierarchical microporous/mesoporous zeolites. In this method, a linear fit at low thickness of adsorbate film (low relative pressure) provides the micropore volume *V*_micro_ (the intercept) and the mesopore plus external surface area *S*_meso+ext_ (the slope). Then the micropore surface area can be roughly estimated as follows [38]: *S*_micro_ = *S*_BET_ − *S*_meso+ext_. An example of *t*-plot analysis is shown in Figure 5e. The pore volumes and surface areas evaluated from *t*-plot are also listed in Table 2.

**Table 2:** Textural properties of the Ti-loaded samples from N_2_ adsorption isotherms.

Sample	*S*_BET_ (m^2^/g)	*S*_meso+ext_ (m^2^/g)	*S*_micro_ (m^2^/g)	*V*_BJH_ (cm^3^/g)	*V*_micro_ (cm^3^/g)	*D*_BJH_ (nm)

Ti-W6h-C	134	131	3	0.17	0.004	3.8
Ti-W12h-C	178	178	0	0.22	0.005	4.0
Ti-W24h-C	178	170	8	0.19	0.007	4.1
Ti-E6h-C	268	226	42	0.29	0.022	4.1/6.0^a^
Ti-E12h-C	183	179	4	0.26	0.005	4.1/7.2^a^
Ti-E24h-C	259	241	18	0.29	0.015	4.1/6.0^a^

^a^For the Ti-E*N*h-C series two pore diameter values correspond to two maxima in the pore size distribution curves.

According to the *t*-plot analysis, the contribution of micropores to the total pore volume does not exceed 10% and is even lower for the sample hydrolyzed in water. As can be seen from Figure 5c, hydrolysis in water leads to the formation of mesopores of about 3.8 nm in size with a mesopore volume of 0.17 cm^3^/g. Increasing the hydrolysis time up to 12 h results in a slight increase of mesopore size and volume (both evaluated from BJH and *t*-plot methods) that correlates with a more developed surface area. However, prolongation of hydrolysis time does not affect the textural properties of the resulting composites. With an increase hydrolysis duration in the presence of ethanol, first, there is a sharp decrease in porosity. Then, there is an increase to almost the same value as it was after the minimum processing time, which may be due to the formation of microporous zeolite. The decrease in specific surface area for Ti-E12h-C compared to Ti-E6h-C is most likely due to the formation of larger mesopores, which are not observed in Ti-E6h-C and T-E24h-C.

It is interesting to note that the surface area of the studied composites obtained by pillaring of lamellar mordenite with anatase nanoparticles formed in the interlamellar space via hydrolysis is very close to the one reported for TiO_2_/ZSM-5 composites obtained by a related method (by adding the presynthesized zeolite in the synthesis medium of TiO_2_ and, vice versa, by adding presynthesized TiO_2_ in the synthesis medium of zeolite [27]), lower than in a mechanical mixture of TiO_2_ with microporous zeolite, but higher than for composites obtained by a liquid impregnation method [40].

**Figure 6:**
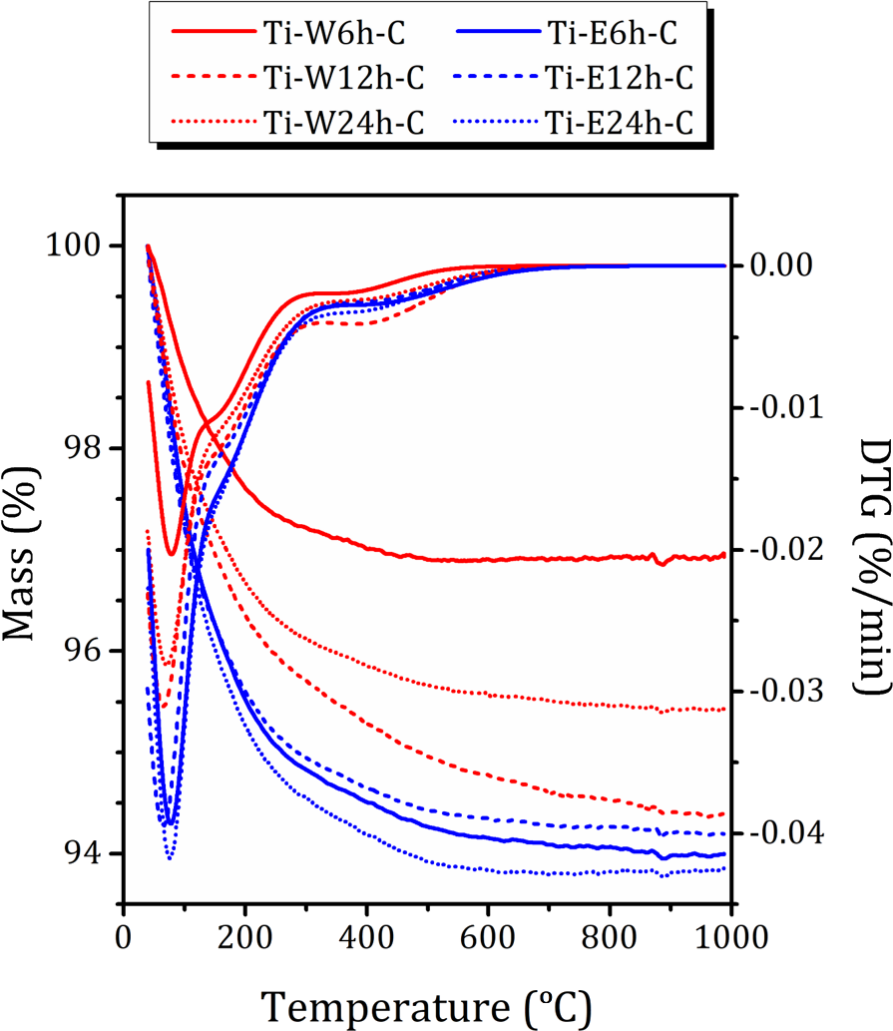
TG and DTG profiles for the studied nanocomposites.

Thermogravimetric (TG) profiles of the studied samples together with the derivative thermogravimetric (DTG) curves are shown in Figure 6. As can be seen, mass loss occurred in two main steps, namely, the rapid desorption of surface water or weakly bound water molecules in the temperature range of 40–100 °C and further mass loss in the temperature range of 100–300 °C, attributed to desorption of the remaining water enclosed in voids and channels [41–42]. However, even at temperatures above 300 °C, all samples exhibit a small peak in the DTG curves at about 400 °C, which can be attributed either to water molecules trapped in hardly accessible voids or, more probably, to a removal of specific hydroxy groups [34]. Moreover, all samples except Ti-W4h-C show a linear mass loss starting from 300 °C that is not reflected by DTG and can be attributed to progressive dehydroxylation (for hydroxy groups with a significant inhomogeneity) [41]. For the sample Ti-W12h-C, this effect is most pronounced and corresponds to about 7.6% of the total mass loss. The total mass loss Δ*w* as well as the mass loss below and above 300 °C (Δ*w*_1_ and Δ*w*_2_, respectively) are listed in Table 3. It should be noted that the samples contain different amounts of TiO_2_ (Table 1). Water is mainly located in zeolite voids. Hence, for a more appropriate determination of the amount of water by mass loss, we need to take it into account. The mass losses below and above 300 °C, recalculated assuming that they are due to the zeolite phase alone, Δ*w*_1_′ and Δ*w*_2_′, respectively are also listed in Table 3.

**Table 3:** Mass loss below (Δ*w*_1_, Δ*w*_1_′) and above (Δ*w*_2_, Δ*w*_2_′) 300 °C as directly determined from TG curves (Δ*w*_1_, Δ*w*_2_) and recalculated accounting for TiO_2_ content (Δ*w*_1_′, Δ*w*_2_′). The total mass losses Δ*w* and Δ*w*′ are also provided.

Sample	40 °C < *T* < 300 °C		300 °C < *T* < 1000 °C		300 °C < *T* < 1000 °C	

	Δ*w*_1_ (%)	Δ*w*_1_′ (%)	Δ*w*_2_ (%)	—	Δ*w*_1_ (%)	Δ*w*_1_′ (%)

Ti-W6h-C	2.8 ± 0.1	7.6 ± 0.3	0.3 ± 0.1	Ti-W6h-C	2.8 ± 0.1	7.6 ± 0.3
Ti-W12h-C	3.9 ± 0.1	14.4 ± 0.3	0.7 ± 0.1	Ti-W12h-C	3.9 ± 0.1	14.4 ± 0.3
Ti-W24h-C	4.3 ± 0.1	15.3 ± 0.3	1.3 ± 0.1	Ti-W24h-C	4.3 ± 0.1	15.3 ± 0.3
Ti-E6h-C	5.2 ± 0.1	13.3 ± 0.5	0.9 ± 0.1	Ti-E6h-C	5.2 ± 0.1	13.3 ± 0.5
Ti-E12h-C	5.0 ± 0.1	14.8 ± 0.3	0.8 ± 0.1	Ti-E12h-C	5.0 ± 0.1	14.8 ± 0.3
Ti-E24h-C	5.5 ± 0.1	10.9 ± 0.3	0.7 ± 0.1	Ti-E24h-C	5.5 ± 0.1	10.9 ± 0.3

As one can see, there is now obvious correlation between water content determined from Δ*w*_1_′ and the mesopore volume *V*_BJH_ estimated from nitrogen adsorption. Despite the larger pore volume and surface area, there is about the same amount of water in the samples obtained by hydrolysis in 70% ethanol solution and in pure water. Nevertheless, there is a strong correlation between the water content Δ*w*_1_′ and the amount of hydroxy groups, Δ*w*_2_′. This points out to a strong interaction of nanoconfined water with hydroxy groups on the inner surface of mesopores, a part of which formed upon calcination [6]. Moreover, the samples obtained in 70% ethanol solution may exhibit different hydrophilic/hydrophobic properties than the samples hydrolyzed in pure water. It is known that the hydrophilicity of TiO_2_ depends on the crystalline phase and surface composition, but not on the size [43]. Moreover, it was found that introduction of TiO_2_ into an amorphous siliceous matrix increases the hydrophilicity of the material. However, as it was shown in [44], the formation of a dense anatase phase has a strong influence on both the value of water adsorption energy and the distribution of water adsorption centers.

### UV–vis spectrometry

To determine the bandgap energy *E*_g_, the Tauc method was applied to the diffuse reflectance spectra. In this method, it is assumed that the energy-dependent absorption coefficient α can be written as


[4] $${\left(\alpha \cdot h\nu \right)}^{1/n}=B\left(h\nu -E_{\text{g}}\right),$$

where *h* is the Planck constant, *h*ν is the photon energy, and *B* is a constant. The factor *n* depends on the nature of the electron transition, that is, *n* = 1/2 for direct and *n* = 2 for indirect transition bandgaps. The Kubelka–Munk method allows one to transform the reflectance spectra into the corresponding adsorption spectra using the function


[5] $$F\left(R\right)=\frac{K}{S}=\frac{{\left(1-R\right)}^{2}}{2R},$$

where *R* is the reflectance of a thick uniform sample, and *K* and *S* are the adsorption and scattering coefficients, respectively. When *F*(*R*_∞_) ∼ α, by combing Equation 4 and Equation 5, one obtains


[6] $${\left(F\left(R\right)\cdot h\nu \right)}^{1/n}=B\left(h\nu -E_{\text{g}}\right).$$

**Figure 7:**
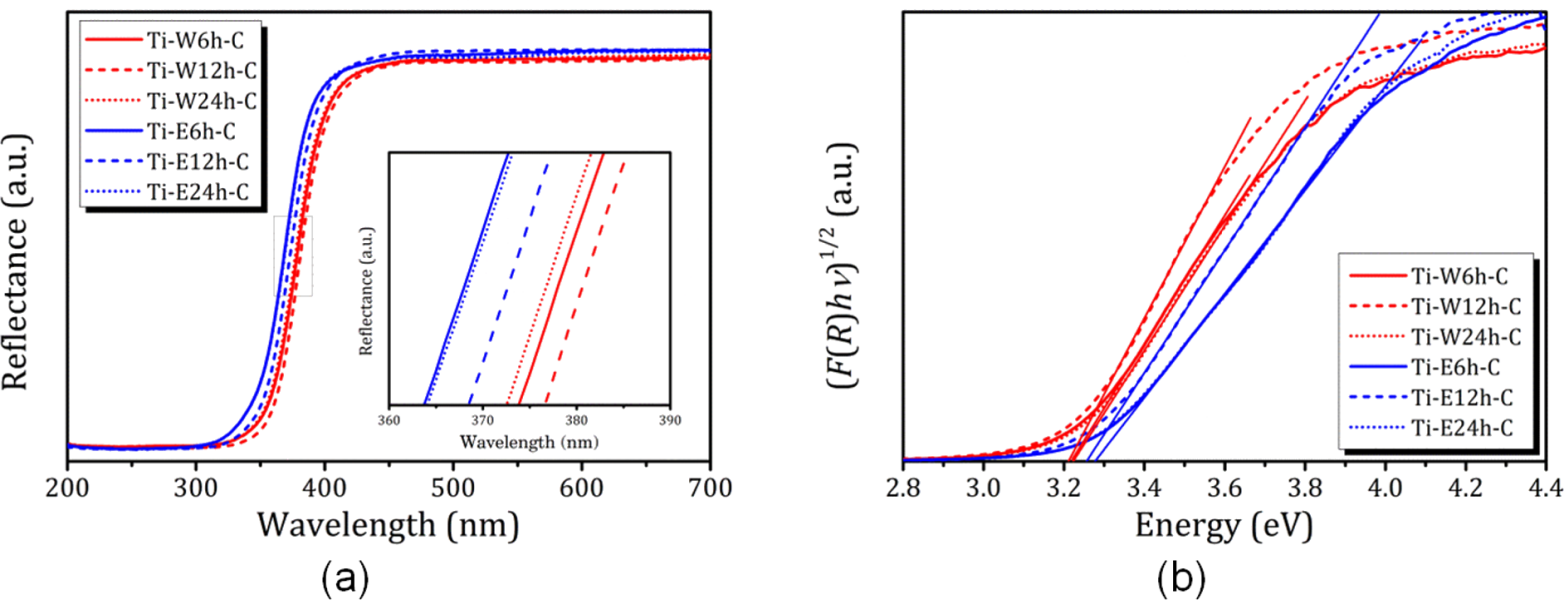
(a) UV–vis diffuse reflectance spectra and (b) plots of (*F*(*R*)*h*ν)^1/2^ versus photon energy for calculation of bandgap energies of TiO_2_-loaded mordenite.

Figure 7a shows the diffuse reflectance spectra of the prepared TiO_2_/2D mordenite nanocomposite. All samples exhibit absorption edges near 400 nm due to the anatase TiO_2_ bandgap absorption. Figure 7b shows the reflectance spectra of the studied nanocomposites transformed using Equation 6 with *n* = 2 (since TiO_2_ is an indirect bandgap semiconductor). Semiconductor materials are characterized by a steep linear increase in light absorption with increasing energy. The bandgap energy can be estimated from the point of intersection of the *x*-axis of the linear fit of the Tauc plot.

As one can see from Figure 7b, the *E*_g_ values for the studied TiO_2_/2D mordenite nanocomposites are sensitive to the hydrolysis medium. The samples obtained by hydrolysis in water exhibit *E*_g_ values of about 3.22 eV, which is close to the value of bulk anatase [45–46]. Hydrolysis in 70% ethanol solution results in higher *E*_g_ values, about 3.27 eV, which are typical for anatase nanoparticles [45].

It should be noticed that according to a number of studies, the bandgap width in TiO_2_/zeolite composites depends both on the topology of the zeolite framework [47] and on the ratio between TiO_2_ and zeolite [47–48]. Alvarez et al. [47] reported that for TiO_2_ deposited on mordenite during the TiO_2_ sol–gel synthesis in a ratio of 75:25, which is close to that in our work (i.e., between 50:50 and 70:30), leads to a noticeable increase in the bandgap (3.47 eV) [47]. It should also be noted that TiO_2_/zeolite composites that were obtained by either incorporating the presynthesized zeolite in the synthesis medium of TiO_2_ or incorporating presynthesized TiO_2_ in the synthesis medium of ZSM-5 [27] exhibit optical properties very similar to those found in our work.

## Conclusion

A series of pillared TiO_2_/2D mordenite nanocomposites were successfully synthetized from the initial lamellar CTAB/2D mordenite by introducing TEOT, hydrolyzing the resulting composite in either water or 70% ethanol solution with hydrolysis reaction times of 6, 12, and 24 h, and subsequent calcination. The main results of the comprehensive study of the obtained composites can be summarized as follows. (1) A study of the TEOT hydrolysis process was carried out, and it was found that the properties of the samples depend significantly on the hydrolysis medium and the duration of the process. The hydrolysis of TEOT in the interlamellar CTAB layers in all cases disrupts the long-range ordering of zeolite lamellae. Ethanol allows one to attenuate and slow down this process compared to pure water. In samples prepared in 70% ethanol solution, the residual ordering of the lamellar structure at the first stage of the process is preserved to some extent, and the textural characteristics are better compared to samples prepared in pure water. Further calcination, regardless of the medium in which TEOT hydrolysis occurred, leads to the final disappearance of the long-range ordering of zeolite lamellas. (2) According to XRD data, anatase nanoparticles of about 4 nm in size form pillars separating the mordenite layers, creating the mesoporosity observed in the experiment. The textural properties of the samples strongly depend on the hydrolysis medium. Hydrolysis in 70% ethanol solution leads to a bimodal distribution of pore sizes, with peaks at 4 and 6 nm, as well as to an increase in the volume of mesopores. (3) Hydrolysis in 70% ethanol solution for 24 h leads to recrystallization of 2D mordenite and to the formation of thin single-crystalline fibers of 3D mordenite. (4) Immobilization of TiO_2_ nanoparticles on the zeolite support was confirmed by XPS. (5) Samples obtained by hydrolysis in water exhibit a bandgap of 3.22 eV, which is close to that of bulk anatase. Hydrolysis in 70% ethanol solution results to higher bandgap values, around 3.27 eV, which are characteristic of anatase nanoparticles.

Despite the still large bandgap, the immobilization of TiO_2_ on the zeolite matrix, combined with the mesopore structure important for high mass transfer properties, suggests that these materials may be promising catalysts under flow conditions. However, it is necessary to further search for parameters to regulate the growth of nanoparticles in the interlayer space (e.g., the temperature of hydrolysis) and their morphology, as well as to investigate the influence of synthesis conditions on the target properties. Nevertheless, the successful application of zeolite pillaring using an oxide material other than SiO_2_ opens up a wide range of opportunities for the development of new materials for specific applications.

## Experimental

The synthesis of nanocomposites was carried out similarly to the method first proposed in [49] and used previously in [5,50] for the insertion of SiO_2_ nanopillars. The latter includes the following steps: (1) synthesis of layered zeolite by self-assembly using CTAB as an organic structural guiding agent that creates regular mesopores, (2) introduction of TEOS molecules into the interlayer space, (3) hydrolysis and formation of pillars of amorphous SiO_2_ separating the layers of two-dimensional zeolite, and (4) calcination for the removal of organic molecules. In this work, to obtain TiO_2_/2D mordenite composites (layers of two-dimensional mordenite separated by TiO_2_ pillars), the synthesis method was slightly adjusted and TEOS was replaced by TEOT. This substitution, taking into account the differences in the properties of these compounds, required changes in both the reagents for hydrolysis (processes in pure water and in 70% ethanol solution were compared) and in the choice of process time intervals.

In the synthesis of TiO_2_/2D mordenite compounds, the layered MOR-L sample obtained in step (1), was stirred in TEOT at a mass ratio of 1:5 for 6 h at 25 °C. The sample was filtered off and then dried at 35 °C for 12 h. To hydrolyze TEOT that had diffused into the CTAB layers and to obtain TiO_2_ nanoparticles in the interlamellar space, 1.0 g of TEOT-impregnated MOR-L sample was stirred in either 10.0 g of distilled water or a 70% ethanol-in-water solution, at 90 °C, for 6, 12, and 24 h. After completion of the hydrolysis, the samples were filtered, washed with distilled water, dried at 120 °C, and finally calcined at 550 °C for 4 h in air. The calcined (C) samples are labeled as Ti-W*N*h-C and Ti-E*N*h-C with *N* = 6, 12, and 24 for materials hydrolyzed in water (W) and 70% ethanol solution (E) for 6, 12, or 24 h, respectively. The non-calcined samples are labeled as Ti-W*N*h and Ti-E*N*h.

Powder XRD analysis was carried out on a Bruker D8 DISCOVER diffractometer with monochromatic Cu Kα radiation. XRD patterns of the studied compounds were recorded in the 2θ range of 5–70° with a step width of 0.0302°. SAXS patterns were recorded over a 2θ scanning range of 0.2–7.0° with a step width of 0.01°.

Elemental analysis and morphology of synthetized TiO_2_/2D mordenite nanocomposites were probed by SEM-EDX using a Zeiss Merlin microscope equipped with an Oxford Instruments INCAx-act EDX console.

N_2_ sorption isotherms were recorded at 77 K using a QuadraSorb SI instrument. Before analysis, samples were outgassed under vacuum for 6 h at 300 °C. The surface areas were estimated within the mBET method. Thermal gravimetric analysis (TGA) was carried out using a Netzsch STA 449 F1 Jupiter instrument in the temperature range of 40–990 °C at a heating rate of 10 °C/min in an Ar flow of 90 mL/min.

^27^Al MAS NMR spectra were recorded using a Bruker Avance IIIWB 400 MHz solid-state NMR spectrometer (operating with Topspin version 3.2) using a double-resonance 4 mm MAS probe with a rotor speed of 12.5 kHz.

XPS spectra of the samples were taken using a Thermo Fisher Scientific Escalab 250Xi spectrometer with non-monochromatic Al Kα radiation (photon energy 1486.6 eV). Bandgaps energies were determined by diffuse reflectance spectroscopy using a Lambda 1050 spectrophotometer (Perkin Elmer) equipped with an integrating sphere in the spectral range of 200–700 nm. Barium sulfate (BaSO_4_) was used as a reference.

## Data Availability

The data that supports the findings of this study is available from the corresponding author upon reasonable request.
